# Sustainable Material Selection for Interior Design Furniture: A Simple Procedure Based on Environmental Analysis and Structural Optimization

**DOI:** 10.3390/ma18092023

**Published:** 2025-04-29

**Authors:** Paolo Trucillo, Farah Chaouali, Francesco Paolo Antonio Portioli

**Affiliations:** 1 Dipartimento di Ingegneria Chimica, dei Materiali e della Produzione Industriale, University of Naples Federico II, Piazzale Vincenzo Tecchio, 80, 80125 Naples, Italy; 2 Department of Structures for Engineering and Architecture, University of Naples Federico II, Via Forno Vecchio, 36, 80134 Naples, Italy; f.chaouali@studenti.unina.it (F.C.); fportiol@unina.it (F.P.A.P.)

**Keywords:** sustainable materials, structural optimization, Finite Element Analysis (FEA), furniture design, material selection, environmental impact, cost efficiency

## Abstract

A simple procedure is proposed for the sustainable selection of materials for interior design. The procedure is based on structural, environmental, and material cost analysis. For structural analysis, finite element models are used to analyze the behavior in terms of stress and strains and to optimize the product size. For environmental analysis, the assessment focuses on the carbon footprint of each material, considering CO_2_ emissions throughout its lifecycle. To show the potentialities of the proposed procedure, the Iso-Lounge chair by Jasper Morrison was selected as a case study. The research evaluates three materials (plywood, polypropylene, and polycarbonate), assessing their mechanical properties, cost implications, and CO_2_ emissions. Results indicate that plywood, with a reduced thickness in the redesigned model, maintains structural integrity while significantly lowering the amount of material used. Compared to polypropylene and polycarbonate, which required greater thickness, plywood demonstrated superior mechanical efficiency and cost-effectiveness. Additionally, CO_2_ emissions analysis revealed that plywood had a lower footprint than polycarbonate and was comparable to polypropylene. These findings highlight the advantages of engineered wood in sustainable furniture design, offering a balance between performance, affordability, and environmental responsibility. The redesign approach optimizes material use, demonstrating the potential for reducing waste and enhancing sustainability in structural applications.

## 1. Introduction

Furniture safety is an important aspect for interior design, yet it is often underestimated [1]. According to a study by the U.S. Consumer Product Safety Commission (CPSC), furniture-related accidents accounted for approximately 18,000 injuries and 100 fatalities annually in the United States alone, primarily due to tipping and structural failures [2].

Global research shows that unstable and weak furniture, especially chairs, significantly increases the risk of musculoskeletal injuries and workplace hazards [3,4,5]. The environmental impact of furniture manufacturing is substantial, with the industry responsible for approximately 5% of global deforestation and generating over 9 million tons of waste per year [6,7,8,9]. To address these challenges, regulatory bodies such as the European Committee for Standardization (CEN) and the American National Standards Institute (ANSI) have established rigorous safety and sustainability standards, ensuring that furniture meets the specific requirements [10].

Beyond stability and structural integrity, furniture design must ensure user safety and well-being, particularly in high-risk environments such as healthcare settings. Evidence-based design (EBD) research shows that poorly designed furniture can harm health, even aiding the spread of infections due to hard-to-clean surfaces. This concern is even more critical when designing furniture for elderly and disabled individuals, who are more vulnerable to accidents and discomfort caused by inadequate ergonomics and insufficient structural support. Studies in inclusive and assistive design underline the importance of stability, ease of access, and proper load distribution to ensure safety and independence. Furniture tailored for these populations must meet stricter criteria for comfort, durability, and functionality, making material selection and design optimization essential aspects of the development process [11,12,13]. A study by Malone and Dellinger (2011) found that inappropriate furniture choices in hospitals increased the likelihood of patient harm, while ergonomic and sustainable design solutions significantly reduced injury rates and improved user comfort [14,15]. These findings align with broader concerns in interior design, where safety, durability, and environmental sustainability must converge to create functional and risk-free living and working spaces. Consequently, regulatory frameworks and standards are evolving to integrate both safety requirements and sustainability criteria in furniture manufacturing, ensuring that design choices not only enhance aesthetics but also minimize potential hazards [16].

Different standards regulate the safety of products, providing specifications for requirements such as stability, strength, and durability. For stability, seating shall not overturn under prescribed (or required) values of vertical and horizontal forces, applied on the seat and the back, respectively. Values of vertical and horizontal forces depend on the type of verification, for example, forward or backward overturning. Similar requirements are provided for strength and durability verifications. In those cases, different loads and numbers of cycles are considered to reproduce suitable service and ultimate conditions. According to these standards, verifications are carried out by testing, using appropriate procedures that are detailed for different verifications. Testing procedures include specifications of loading points, loading devices, and supports. To support the design in the preliminary phases and before testing methods to qualify products, numerical analysis can be performed to optimize the structural behavior in terms of stress and displacement. Different studies using finite element models to support the design of furniture, such as a chair, were presented in the literature [17,18,19]. These studies highlight the advantages of FEA in early-stage furniture design. For example, Song et al. (2024) used finite element simulations to validate the structural integrity of thin-walled plastic chairs [17], optimizing thickness distribution to reduce material usage without compromising strength. Mahantesh et al. (2023) demonstrated how FEA supports ergonomic assessments in seated postures, contributing to enhanced comfort and injury prevention [18]. Similarly, Suarez et al. (2021) explored the structural behavior of cardboard-based seating, showing that single-material designs can achieve satisfactory performance through geometric optimization [19]. These findings underscore the relevance of FEA as a predictive and cost-effective tool in the furniture design process, particularly when evaluating different materials and configurations prior to physical prototyping.

In this study, a simple integrated procedure is proposed that relies on structural and environmental criteria to guide the selection and optimization of materials for furniture design. The methodology combines Finite Element Analysis (FEA) to evaluate mechanical performance with environmental impact assessment based on carbon footprint calculations. The structural optimization aims to reduce material usage while maintaining mechanical integrity, ensuring that the redesigned product meets the required safety and performance standards. Environmental assessment quantifies CO_2_ emissions associated with different material choices, providing a comparative framework for sustainable selection. To demonstrate the effectiveness of this approach, the iso-lounge chair by Jasper Morrison was chosen as a case study. Three materials (plywood, polypropylene, and polycarbonate) were compared and analyzed to evaluate their structural behavior, cost implications, and environmental footprint. The iso-lounge chair was chosen as a case study due to its iconic design, minimalist structure, and efficient use of engineered wood. This chair represents an ideal benchmark for sustainable furniture design, as it combines visual simplicity with technical complexity. Its layered plywood construction involves localized reinforcement and material thinning, making it particularly suitable for simulation-based structural analysis. The paper will present a methodology for sustainable material selection and will discuss results from structural and environmental analyses.

## 2. Proposed Approach to Sustainable Design

Sustainable design requires a comprehensive and exhaustive evaluation, integrating economic, environmental, and social impacts of a specific design project. This paper outlines a systematic approach to a design based on sustainability indicators, as proposed by Trucillo et al. in previous studies on materials and processes [20,21]. A sustainable material or process is one that not only ensures profitability and efficiency but also minimizes ecological footprints and considers human well-being. To achieve this, sustainability indicators serve as quantifiable metrics that assess various aspects of sustainability, from carbon emissions to cost efficiency and social acceptance. These indicators are normalized using a percentage-based scale, allowing for a fair and comparative evaluation of design alternatives.

The novelty of this approach lies in its fusion of Finite Element Analysis (FEA) with normalized sustainability scoring. The methodology unfolds in the following stages: (1) material screening and characterization, i.e., selection of candidate materials based on mechanical properties (e.g., elastic modulus, yield strength), environmental data (e.g., CO_2_ emissions, water intensity), cost, and recyclability potential; (2) finite element simulation and structural optimization, i.e., the chair design is modeled in CAD and simulated in Abaqus using shell elements (S4R). Mechanical constraints such as maximum stress and allowable displacement are applied. For each material, the minimum thickness required to meet safety standards is identified, enabling material-saving geometries; (3) quantification of Sustainability Indicators, such as key indicators—mass, cost, CO_2_ emissions, water use, recyclability, normalized as percentages; (4) multicriteria decision analysis with radar diagrams. The normalized scores are plotted in radar diagrams to visualize trade-offs between design options. This supports intuitive and data-driven decision-making, allowing the designer to select materials and geometries that maximize performance; and (5) iterative refinement: If initial configurations do not yield satisfactory trade-offs, the process is repeated with adjusted design parameters or alternative materials. This loop continues until an optimal solution is identified.

This proposed approach aims to enhance decision-making in material selection, not only in process optimization, but also in the selection of proper materials for design applications. With the use of this methodology, designers can direct their choice by comparing different design choices, showing the way for more responsible industrial practices. Details and formulas for the transformation of indicators into dimensionless and comparable variables are explained in Section 4.5.

**Figure 1:**
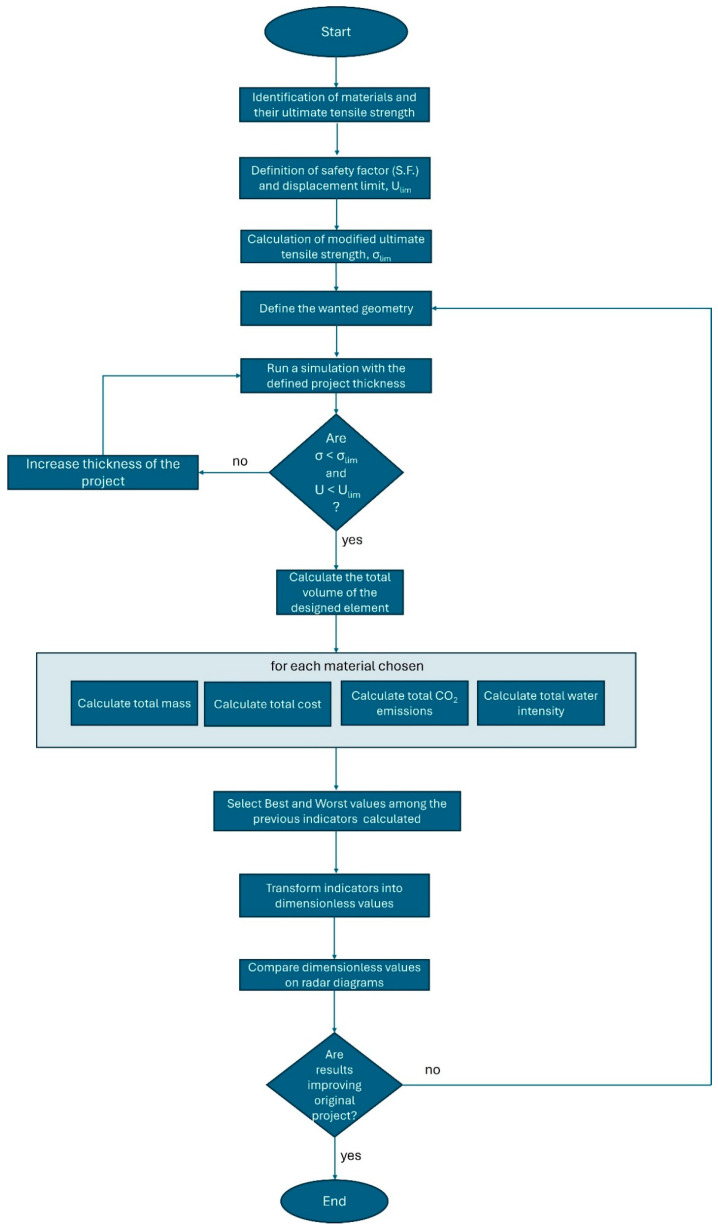
A flux diagram describing the decisional process used for this study.

The flowchart in Figure 1 outlines a structured process for selecting materials and optimizing a project’s design based on mechanical and sustainability criteria. It begins with identifying the materials and their ultimate tensile strength (UTS), followed by defining a safety factor (SF) and displacement limit. The modified ultimate tensile strength is calculated as the UTS divided by the SF; then, the desired geometry is established. A simulation is run with the defined thickness to check whether the calculated stress and the defined displacement are lower than the constraint limits. If they do not, the thickness is increased, and the simulation is repeated; otherwise, the total volume of the designed element is determined. For each selected material, key indicators such as total mass, cost, CO_2_ emissions, and water intensity are calculated. The best and worst values among these indicators are identified, and the indicators are transformed into dimensionless values for comparison. Radar diagrams are used to analyze and compare the materials’ performance. Finally, a decision is made on whether the results improve the original project; if not, the process iterates, and if they do, the optimization is complete. This methodology ensures structural integrity while optimizing economic and environmental impact.

A sketch of the decisional diagram flux has been reported in Figure 1.

## 3. The Case Study

The original chair, iso-lounge, designed by Jasper Morrison, was selected as a case study, simulating the impacts on cost, weight, and sustainability deriving from using different materials.

**Figure 2:**
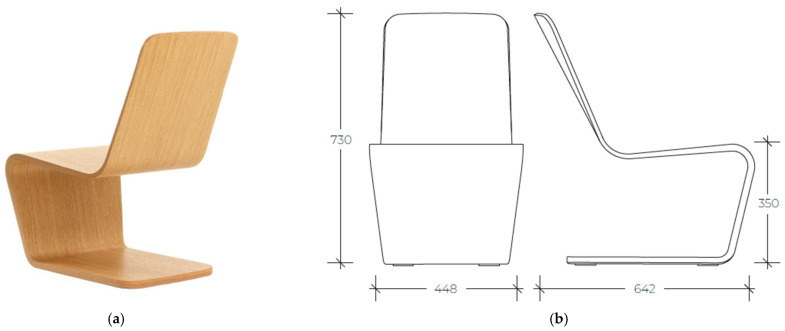
Visual appearance (**a**) and mean dimensions in millimeters (**b**) of the iso-lounge chair.

The iso-lounge chair (Figure 2), though seemingly simple in form, pushes the technical performance of plywood as a material. The plywood layering is reduced in some areas to allow the chair to flex while adding extra layers in areas where the chair requires extra strength and resilience, increasing the chair’s responsiveness to the user. Jasper Morrison and the Isokon Plus team also experimented with the orientation and thickness of the veneer layers. This detailed technical development allowed Morrison to create a slim and sleek silhouette that is incredibly strong. According to the information collected, the plywood shows a significant ability to withstand an applied load without failure or plastic deformation. For the plywood, its bending abilities allow designers to shape it thanks to its excellent steam-bending capability.

Plywood is a wood-based panel made by gluing thin sheets with alternating grain directions, providing strength and resistance to deformation. According to the literature, the plywood shows a significant ability to withstand an applied load without failure or plastic deformation. Moreover, it has several other properties such as resistance to the effects of insects and fungi [22,23,24,25,26].

## 4. Materials and Methods

To study other materials and redesign geometries, the original chair project has been reproduced using the finite element method. Moreover, the software Abaqus v. 2021 (Dassault Systèmes, Paris, France) has been employed to study the distribution of strengths and displacements on the model chair. The three chosen materials were plywood, polypropylene, and polycarbonate, whose mechanical, cost, and environmental data are summarized in Table 1. Elastic modulus and density are well known, but unitary cost, CO_2_ equivalent emissions, and water intensity were obtained by averages from literature research [27,28,29,30].

### 4.1. Plywood

Since plywood does not present a fatigue limit, a compressive strength of 41.8 MPa has been chosen as the constraint limit for the analysis and simulations. An elastic modulus of 8600 MPa and a Poisson ratio of 0.036 were considered for this material, according to the available database (source: Matweb.org). For these simulations, a safety factor of 1.5 has been chosen; therefore, the tensile strength limit of 41.8 MPa has been divided by this factor, thus resulting in a modified tensile strength limit of 27.86 MPa. The displacement limit was fixed at 20 mm to ensure both stability and ergonomic comfort under load. These parameters provide a realistic and cautious basis for evaluating plywood performance under typical seating loads.

### 4.2. Polypropylene

Polypropylene (PP) is a thermoplastic polymer derived from the polymerization of propylene monomers, commonly utilizing Ziegler–Natta catalysts [31]. Its high fatigue resistance, impact strength, and thermal stability make it suitable not only for industrial applications but also for original design projects [32,33,34], including packaging, machinery components, and textiles [35,36,37,38]. PP’s versatility has been harnessed to create iconic seating solutions.

Notably, Robin Day’s 1963 Polypropylene Chair revolutionized mass-produced furniture with its injection-molded PP shell, offering a lightweight, durable, and cost-effective seating option. This design has achieved global ubiquity, with millions produced and utilized in diverse settings. Further exemplifying PP’s application in design, the Picto chair, introduced by Hans Roericht in 1993, features a fully dismountable structure composed of polypropylene. This design emphasizes recyclability and environmental considerations, aligning with contemporary sustainability goals. These instances underscored polypropylene’s significant role in modern furniture design, offering a combination of functionality, aesthetic appeal, and environmental consciousness.

An elastic modulus of 1325 MPa and a Poisson ratio of 0.43 were considered for this material, according to the available database (source: Matweb.org). For these simulations, a safety factor of 1.5 has been chosen; therefore, the tensile strength limit of 24 MPa has been divided by this factor, thus resulting in a modified tensile strength limit of 16 MPa. The displacement limit has been set at 20 mm, also in this case.

### 4.3. Polycarbonate

Polycarbonate (PC) is a transparent thermoplastic material. Its high strength makes it resistant to impact and fracture, and it is widely used for its eco-friendly processing and recyclability [39,40,41]. Polycarbonates are strong, stiff, hard, tough, transparent engineering thermoplastics that can maintain rigidity up to 140 °C and toughness down to −20 °C or special grades even lower [42]. The material is amorphous (thereby displaying excellent mechanical properties and high dimensional stability), is thermally resistant up to 135 °C, and is rated as slow burning [43]. Constraints to the use of PC include limited chemical and scratch resistance, and its tendency to yellow upon long-term exposure to UV light. However, these constraints can be readily overcome by adding the right additives to the compound or processing through a coextrusion process [44,45].

An elastic modulus of 2380 MPa and a Poisson ratio of 0.36 were considered for this material, according to the available database (source: Matweb.org). For these simulations, a safety factor of 1.5 has been chosen; therefore, the tensile strength limit of 18 MPa has been divided by this factor, thus resulting in a modified tensile strength limit of 12 MPa. The displacement limit has been set at 20 mm for this final comparison.

### 4.4. The Finite Element Model

A CAD model of the chair was generated using surfaces, which were partitioned on the basis of the different parts of the chair, i.e., the back, the seat, and the support, in order to apply different loads and boundary conditions. The geometric model was imported into Abaqus software to perform the finite element analysis (FEA) for the design of the iso-lounge chair. Shell elements (S4R) were used to mesh the model. Elastic behavior was considered for different materials, with elastic modulus and Poisson ratios corresponding to the values reported in previous sections. Concerning the load, uniformly distributed pressures were applied, corresponding to the load of 2000 N on the seat and 700 N on the back, according to loads indicated for test methods in the standards [46]. For boundary conditions, an encastre was considered at the bottom of the chair.

### 4.5. Sustainability Indicators Calculations

The idea of using an alternative analysis to the traditional life cycle assessment has already been proposed in the literature [47,48]. Sustainability indicators are quantitative or qualitative metrics used to assess, monitor, and communicate progress towards sustainability goals in environmental, social, and economic dimensions. These indicators provide measurable insights into how well a system, organization, or society is performing concerning sustainable development. These indicators are used to compare the scores of design projects in terms of mass, cost, water intensity, mechanical properties, and carbon dioxide equivalent emissions. These indicators are normally characterized by different units of measure. Therefore, to compare them, they were transformed into percentages, named scores. Each score has been calculated considering the range of best-worst, in which the best is the most favorable situation, while the “worst” represents the least sustainable case. As indicated in Equation (1), the scores were calculated as follows:

(1) $$score,\;\%=\frac{Actual-Worst}{Best-Worst}$$

where Actual is the current value of the variable that needs to be transformed into dimensionless percentage. Once calculated, scores were depicted in a radar diagram to visualize the most sustainable solution.

## 5. Results

### 5.1. Original Project: Use of Plywood

**Figure 3:**
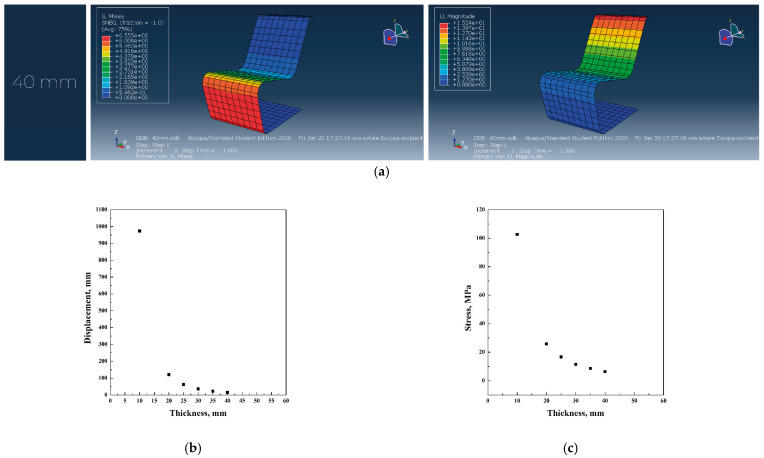
Abaqus finite element analysis describing plywood original project (**a**), displacement vs. thickness (**b**), stress vs. thickness (**c**) diagrams.

In Figure 3a–c, the main simulation results have been reported.

### 5.2. Original Project: Use of Polypropylene

**Figure 4:**
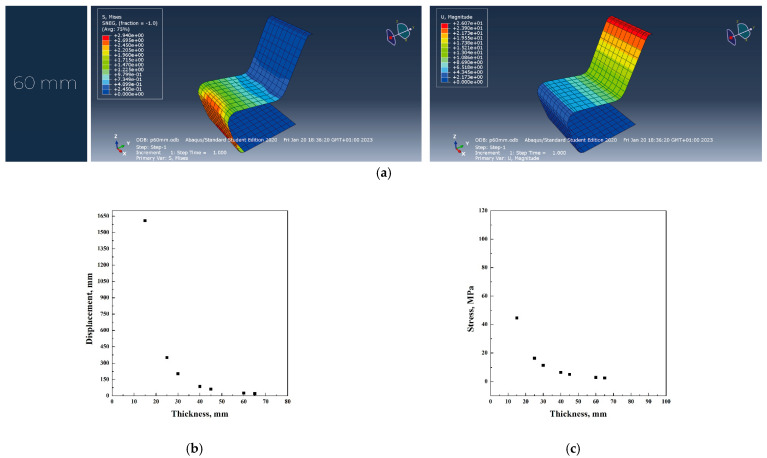
Abaqus finite element analysis describing PP original project (**a**), displacement vs. thickness (**b**), stress vs. thickness (**c**) diagrams.

PP was evaluated as a potential alternative to plywood. However, the study indicates that the use of polypropylene necessitates a significant increase in material thickness to ensure structural integrity and safety (Figure 4a–c). Specifically, while the original design requires a thickness of 40 mm, polypropylene needs a thickness of 60 mm to achieve comparable levels of strength and stiffness. From a mechanical perspective, polypropylene exhibits good resistance to fatigue and impact; however, it demonstrates lower long-term load-bearing capacity compared to wood. To prevent structural failure, the study adopted a fatigue strength value of 24 MPa, which further influenced the required thickness.

### 5.3. Original Project: Use of Polycarbonate

**Figure 5:**
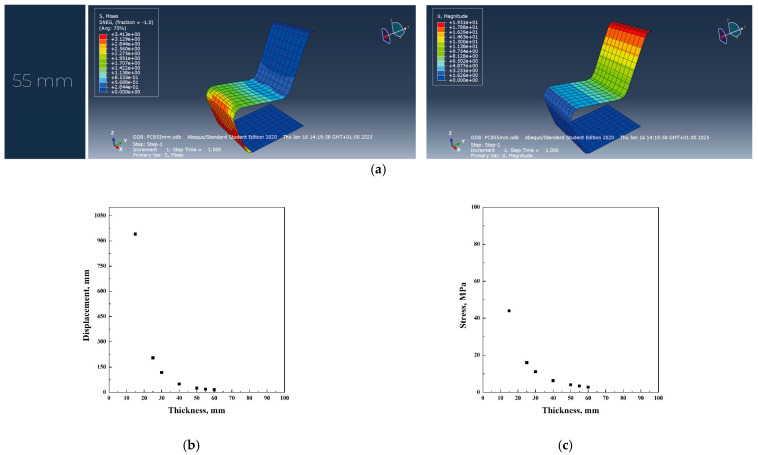
Abaqus finite element analysis describing PC original project (**a**), displacement vs. thickness (**b**), stress vs. thickness (**c**) diagrams.

PC was analyzed as a second alternative material to plywood, offering a balance between mechanical strength, impact resistance, and design flexibility. PC, being a high-performance thermoplastic, is widely used in structural applications. However, its mechanical behavior and cost introduce challenges when compared to the original engineered plywood. Its intermediate rigidity with PC and plywood allows for moderate flexibility while maintaining structural integrity. In this study, a compressive strength of 18 MPa has been used, ensuring a balance between mechanical strength and stiffness (Figure 5a–c).

### 5.4. Discussion

The cantilevered structure of the iso-lounge chair relies on a delicate balance of strength and flexibility, which is efficiently achieved through the plywood layering technique. While polycarbonate allows for molding into complex forms, its higher density and required material thickness result in a heavier and less sustainable product. Furthermore, polycarbonate’s susceptibility to UV degradation and scratching may compromise the chair’s durability and aesthetics.

According to the analysis of the chair’s shape using the software Abaqus with different materials, it was noticed that polycarbonate shows better results in terms of stress and displacement compared to polypropylene. However, plywood mechanical properties show a larger stress for the 40 mm thickness original thickness, compared to polycarbonate and polypropylene, which require a thickness of 60 and 55 mm, respectively. In all the simulations, the first thickness value that verified both stress and displacement conditions was used to calculate the total volume of the iso-lounge chair. In particular, the use of plywood resulted in a thickness of 40 mm, causing a stress of 6.5 MPa and a displacement of 15.2 mm; the use of polypropylene resulted in a thickness of 60 mm, related to a stress of 2.5 MPa and a displacement of 20 mm. Polycarbonate material required a thickness of 55 mm, with a stress of 2.5 MPa and a displacement of 20 mm.

## 6. Redesign Results

**Figure 6:**
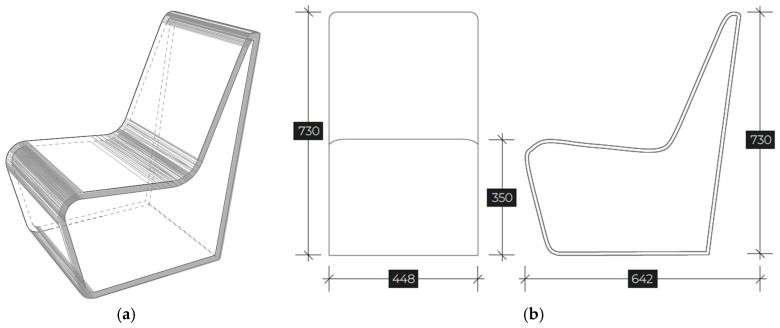
Visual appearance (**a**) and mean dimensions in millimeters (**b**) of the iso-lounge redesigned chair.

In the second part of this study, the iso-lounge chair was redesigned to enhance its structural stability while simultaneously reducing material costs and environmental impact (Figure 6). The redesign focused on optimizing the chair’s geometry and material distribution, ensuring improved mechanical performance without compromising its aesthetic and ergonomic qualities.

The redesign proposed consisted of adding a unique backside panel support, playing a role in the stability of the chair and its overall strength and safety. By refining the structural configuration, the required material thickness was significantly reduced, leading to a lighter yet more stable structure. This optimization allowed for a decrease in material usage, directly impacting both manufacturing costs and carbon emissions. Moreover, Table 2 clearly highlights the reduction in material mass achieved through the redesign. This is particularly evident when comparing the total mass of the original and redesigned versions for each material.

### 6.1. Redesigned Project: Use of Plywood

**Figure 7:**
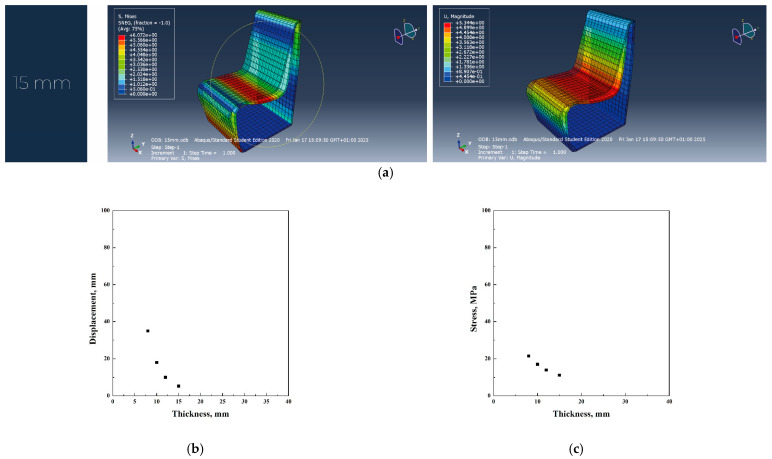
Abaqus finite element analysis describing the oak plywood redesigned project (**a**), displacement vs. thickness (**b**), stress vs. thickness (**c**) diagrams.

The redesign of the iso-lounge chair in plywood aimed to enhance its structural efficiency, reduce material consumption, and improve sustainability while preserving its minimalist aesthetic and ergonomic comfort. By optimizing the plywood layering and overall geometry, the chair maintains its strength and flexibility while significantly reducing the required material thickness (Figure 7a–c).

### 6.2. Redesigned Project: Use of Polypropylene

The redesign of the Iso-Lounge chair in polypropylene aimed to optimize material efficiency, structural performance, and sustainability while maintaining the original chair’s ergonomic and minimalist design. Given polypropylene’s lower stiffness compared to plywood, the redesign focused on adjusting the material thickness and distribution to ensure adequate strength and stiffness.

**Figure 8:**
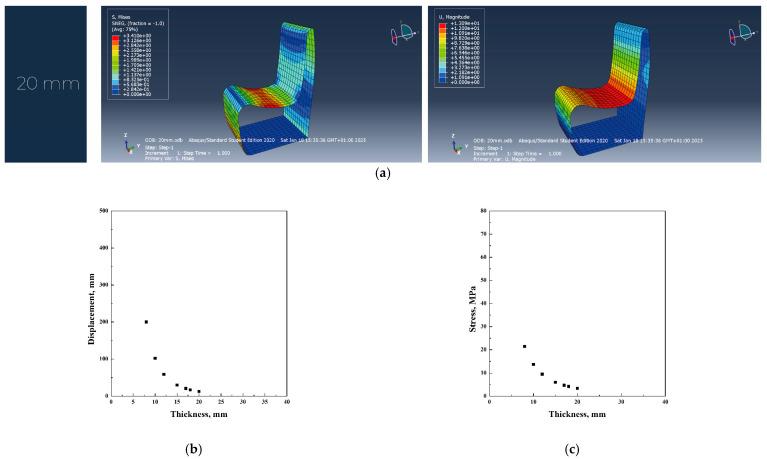
Abaqus finite element analysis describing PP redesign project (**a**), displacement vs. thickness (**b**), stress vs. thickness (**c**) diagrams.

In the original design, polypropylene required a 60 mm thickness to achieve structural stability. However, the redesigned version successfully reduced this to 20 mm, significantly decreasing material usage and weight. This improvement leads to lower production costs and a more sustainable manufacturing process, making polypropylene a more viable option than in its initial analysis (Figure 8a–c).

### 6.3. Redesigned Project: Use of Polycarbonate

Considering polycarbonate’s higher stiffness and impact resistance than polypropylene, the redesign focused on reducing material thickness while ensuring sufficient strength and stiffness.

**Figure 9:**
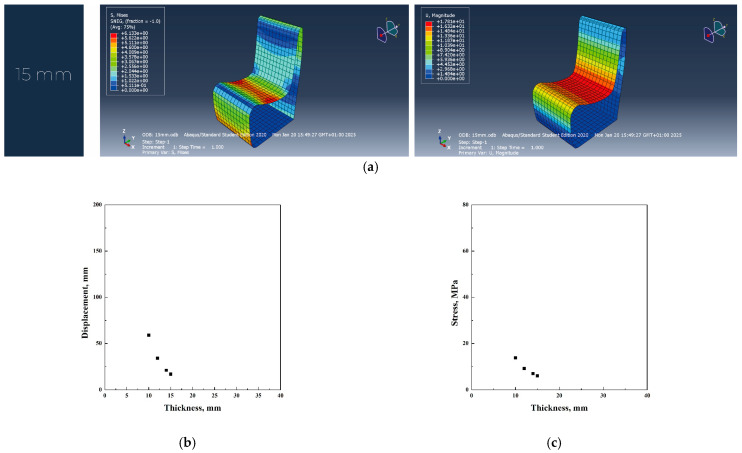
Abaqus finite element analysis describing PC redesign project (**a**), displacement vs. thickness (**b**), stress vs. thickness (**c**) diagrams.

In the original design, polycarbonate required a 55 mm thickness to achieve the necessary load-bearing capacity. However, the redesign successfully reduced this to 15 mm, significantly lowering material consumption and overall weight. This optimization led to reduced production costs and a decrease in CO_2_ emissions, making the redesigned chair a more sustainable alternative than its initial polycarbonate version (Figure 9a–c).

## 7. Calculation of Sustainability Analysis and Comparison

According to the analysis of the redesigned chair on Abaqus, it was possible to note a significant reduction in terms of thickness and weight, also obtaining improved results in terms of stress and displacement than PP. However, also in the case of redesign, plywood simulations showed the lowest thickness (10 mm) compared to 15 and 18 mm required for the other two analyzed materials.

In all the simulations, the first thickness value that verified both stress and displacement conditions was used to calculate the total volume of the iso-lounge chair. In particular, the use of plywood resulted in a thickness of 10 mm, causing a stress of 13.7 MPa and a displacement of 18 mm; the use of polypropylene resulted in a thickness of 18 mm, related to a stress of 4.2 MPa and a displacement of 17 mm. Polycarbonate material required a thickness of 15 mm, with a stress of 6.1 MPa and a displacement of 17 mm.

A set of key indicators was defined to assess each material-geometry combination. These include minimum chair thickness, material mass (linked to raw material use), cost, and environmental impact per unit mass. Mechanical performance, such as stiffness and strength, was also central to the evaluation. Additional criteria considered were water intensity during manufacturing and end-of-life recyclability, distinguishing between mechanical recycling and energy recovery.

**Figure 10:**
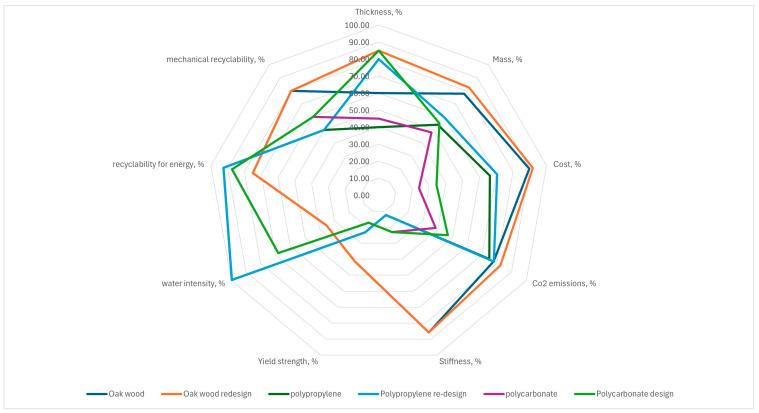
Radar diagram summarizing sustainability indicators for original and redesigned iso-lounge chair configurations across different materials (plywood, polypropylene, polycarbonate).

Therefore, sustainability indicators were calculated starting from the data collected in Table 2. After applying Equation (1), scores in percentages were proposed in Table 3 and shown in the radar diagram of Figure 10.

**Table 2:** Comparison of original and redesign projects varying selected materials (PW: plywood, PP: Polypropylene, PC: Polycarbonate, T: Thickness, WI: water intensity; Y.S.: yield strength).

Material	T, mm	Volume, m^3^	Mass, kg	Cost, €	CO_2_, kg	Stiffness, MPa	Y. S., MPa	W.I., L/kg	Rec. Energy, %	Mech Rec., %
PW	40	0.0314	22.13	15.49	110.65	8600	41.8	645	75	80
PW redesign	15	0.0249	17.49	12.24	87.45	8600	41.8	645	75	80
PP	60	0.0314	45.98	50.57	125.30	1325	24	5.2	92.5	50
PP redesign	20	0.0447	40.25	44.28	109.68	1325	24	5.2	92.5	50
PC	55	0.0432	51.81	113.98	307.54	2380	18	320	87.5	60
PC redesign	15	0.0373	44.73	98.40	265.51	2380	18	320	87.5	60

**Table 3:** Comparison of scores in original and redesign projects (PW: plywood, PP: Polypropylene, PC: Polycarbonate, T: Thickness, WI: water intensity; Y.S.: yield strength).

Material	T, %	Mass, %	Cost, %	CO_2_, %	Stiffness, MPa	Y.S., MPa	W.I., %	Rec. Energy, %	Mech Rec., %
PW	60.00	77.87	89.67	77.87	85.86	41.21	35.54	75.00	80.00
PW redesign	85.00	82.51	91.84	82.51	85.86	41.21	35.54	75.00	80.00
PP	40.00	54.02	66.29	74.94	12.37	23.23	99.58	92.50	50.00
PP redesign	80.00	59.75	70.48	78.06	12.37	23.23	99.58	92.50	50.00
PC	45.00	48.19	24.01	38.49	23.03	17.17	68.07	87.50	60.00
PC redesign	85.00	55.27	34.40	46.90	23.03	17.17	68.07	87.50	60.00

The analysis confirmed that replacing wood with thermoplastic materials generally results in an increase in thickness, weight, and cost. However, the redesigned iso-lounge chair significantly reduced the required project thickness across all the evaluated materials, leading to lower weight, cost, and environmental impact.

Concerning performance evaluation, and observing the scores in Table 3, the plywood Wood redesign emerged as the best-performing configuration, achieving the highest average score of 67.70%. This solution maintains excellent mechanical properties (85.86 MPa stiffness, 41.21 MPa yield strength) while also offering improved cost efficiency and lower CO_2_ emissions compared to the original plywood configuration (61.78%). Additionally, its water intensity is relatively low, reinforcing its sustainability advantages.

The second-best alternative is the polypropylene redesign project, with an overall score of 60.84%. This configuration provided significant improvements in mass and cost efficiency while maintaining similar mechanical performance (12.37 MPa stiffness, 23.23 MPa yield strength) to the original polypropylene version. However, while thermoplastic materials are often marketed as fully recyclable, their mechanical recyclability is limited, as highlighted by the 50% score for PP [49]. This suggests that repeated recycling degrades the material’s mechanical properties, potentially restricting its long-term sustainability.

The original polypropylene configuration scores lower (52.26%), reflecting its higher material consumption and cost. Meanwhile, polycarbonate exhibits the lowest overall performance, with the standard design scoring only 35.24% and the redesigned version 44.65%. Despite offering higher stiffness (23.03 MPa) than polypropylene, polycarbonate is penalized by its high-water intensity (68.07%) and CO_2_ emissions. Although its recovery energy potential is high (87.50%), its mechanical recyclability (60.00%) is still modest, likely due to degradation in polymer chains after multiple reprocessing cycles.

Concerning recyclability, while thermoplastic materials such as polypropylene and polycarbonate are generally recyclable, mechanical recycling alone is insufficient to guarantee a closed-loop system [50]. The quality of recycled thermoplastics degrades with each cycle, meaning that after a few processing loops, the material may require downcycling or incineration for energy recovery rather than being reused for high-performance applications. This limitation significantly impacts their long-term circularity compared to materials like engineered wood, which can be mechanically reprocessed into composite panels without significant loss of structural properties.

Data reported in Table 3 as dimensionless scores were summarized in the radar diagram of Figure 10.

This diagram is the main result of a transformative approach to sustainable furniture design by integrating structural optimization with environmental impact assessment. The proposed methodology allows manufacturers to minimize material usage while maintaining mechanical integrity, significantly reducing costs and carbon emissions. By using Finite Element Analysis (FEA), the study provides a systematic framework for optimizing product design, ensuring that furniture meets stringent safety standards without excessive resource consumption.

The redesign of the iso-lounge chair exemplifies how a data-driven approach can enhance industrial production, yielding lighter, more durable, and cost-effective alternatives without compromising aesthetics or functionality. These findings have direct implications for the furniture manufacturing sector, supporting the adoption of sustainable materials such as engineered wood while demonstrating the potential for thermoplastics in structural applications. By adopting this methodology, industry leaders can improve their environmental footprint, streamline production costs, and align with evolving regulatory and market demands for sustainable design.

## 8. Conclusions

This study evaluated the iso-lounge chair’s structural efficiency, material selection, and sustainability through computational analysis. The findings confirmed that oak plywood offers an optimal balance between mechanical strength, cost-effectiveness, and environmental sustainability compared to alternative thermoplastic materials. The redesign approach significantly improved material distribution, reducing weight and enhancing sustainability performance. These results emphasize the potential of engineered wood as a superior choice for sustainable furniture design.

The results promote sustainable design and circular economy in furniture by encouraging efficient material use. Future studies should validate simulations experimentally and explore advanced materials and design tools to enhance recyclability and reduce waste.

## Figures and Tables

**Table 1:** Main properties of selected materials for simulations (source: Matweb.org).

Material	Elastic Modulus, MPa	Density, kg/m^3^	Unitary Cost, €/kg	CO_2_ Emissions, kg_CO2_/kg	Water Intensity, L/kg
Plywood	8600	704	0.7	5.00	645
Polypropylene	1325	900	1.1	2.72	5.2
Polycarbonate	2380	1200	2.2	5.94	320

## Data Availability

The raw data supporting the conclusions of this article will be made available by the authors on request.

## Abbreviations

The following abbreviations are used in this manuscript:

FEA	Finite Element Analysis
PC	Polycarbonate
PP	Polypropylene
MPa	Megapascal (unit of pressure or stress)
FEAS	Finite Element Analysis Software
ISO	International Organization for Standardization
T	Thickness
V	Volume
ρ	Density
M	Mass
C	Cost
EBD	Evidence-Based Design
SF	Safety Factor
UTS	Ultimate Tensile Strength

## References

[1] Anjum N., Paul J., Ashcroft R. (2005). The changing environment of offices: A challenge for furniture design. Des. Stud..

[2] Suchy A. (2021). Product Instability or Tip-Over Injuries and Fatalities Associated with Televisions, Furniture, and Appliances: 2020 Report.

[3] Bijalwan A., Misra A. (2016). Design and Structural Analysis of Flexible Wearable Chair Using Finite Element Method. Open J. Appl. Sci..

[4] Hall-Andersen L.B., Broberg O. (2014). Integrating ergonomics into engineering design: The role of objects. Appl. Ergon..

[5] Duarte F., Silva G., Lima F., Maia N. Ergonomics guidelines for the design process. Proceedings of the Society of Petroleum Engineers—SPE International Conference on Health, Safety and Environment in Oil and Gas Exploration and Production.

[6] Likotiko E.D., Nyambo D., Mwangoka J. (2017). Multi-agent based IoT smart waste monitoring and collection architecture. arXiv.

[7] Utama D.M., Ardiyanti N., Putri A.A. (2022). A new hybrid method for manufacturing sustainability performance assessment: A case study in furniture industry. Prod. Manuf. Res..

[8] Hartini S., Ciptomulyono U., Anityasari M., Sriyanto M. (2020). Manufacturing sustainability assessment using a lean manufacturing tool: A case study in the Indonesian wooden furniture industry. Int. J. Lean Six Sigma.

[9] Daian G., Ozarska B. (2009). Wood waste management practices and strategies to increase sustainability standards in the Australian wooden furniture manufacturing sector. J. Clean. Prod..

[10] (2023). Furniture—Seating—Determination of Stability.

[11] (2012). Furniture—Seating—Test Methods for the Determination of Strength and Durability.

[12] Beer P., Olenska S., Podobas I., Zbiec M. (2017). Design for AAL integrated furniture for the care and support of elderly and disabled people. Drv. Ind..

[13] Cacciabue P.C., Vella G. (2010). Human factors engineering in healthcare systems: The problem of human error and accident management. Int. J. Med. Inform..

[14] Bhardwaj P. (2015). Furniture Design Features and Healthcare Outcomes. Latest in Healthcare Management.

[15] Malone E.B., Dellinger B.A. (2011). Furniture Design Features and Healthcare Outcomes.

[16] Safin S., Pintus P., Elsen C. (2020). Ergonomics in design and design in ergonomics: Issues and experience in education. Work.

[17] Song L., Yang M., Shen D. (2024). Structural design and experimental verification of a thin-walled plastic chairs based on the finite element method. Sci. Rep..

[18] Mahantesh M.M., Rao K.V.S.R., Chandra A.C.P., Vijayakumar M.N., Nandini B., Prasad C.D., Vasudev H. (2023). Design and modeling using finite element analysis for the sitting posture of computer users based on ergonomic perspective. Int. J. Interact. Des. Manuf..

[19] Suarez B., Muneta L.M., Romero G., Sanz-Bobi J.D. (2021). Efficient design of thin wall seating made of a single piece of heavy-duty corrugated cardboard. Materials.

[20] Trucillo P., Rizzo M., Errico D., Di Maio E. (2025). Social, Economic, and Environmental Impacts of Bio-Based Versus Fossil-Derived Polyethylene Production. Advanced Sustainable Systems. Adv. Sustain. Syst..

[21] Trucillo P., Erto A. (2023). Sustainability Indicators for Materials and Processes. Sustainability.

[22] Kolář T., Rybníček M. (2010). Physical and mechanical properties of Subfossil Oak (Quercus, SP.) wood. Acta Univ. Agric. Silvic. Mendel. Brun..

[23] Büyüksari Ü., As N., Dündar T., Korkmaz O. (2017). Micro-mechanical properties of Oak wood and comparison with standard-sized samples. Maderas Cienc. Tecnol..

[24] Konnerth J., Eiser M., Jäger A., Bader T.K., Hofstetter K., Follrich J., Ters T., Hansmann C., Wimmer R. (2010). Macro- and micro-mechanical properties of red oak wood (*Quercus rubra* L.) treated with hemicellulases. Holzforschung.

[25] Uzcategui M.G.C., Seale R.D., França F.J.N. (2020). Physical and mechanical properties of clear wood from red oak and white oak. Bioresources.

[26] Sonderegger W., Kránitz K., Bues C.T., Niemz P. (2015). Aging effects on physical and mechanical properties of spruce, fir and oak wood. J. Cult. Herit..

[27] Schyns J.F., Booij M.J., Hoekstra A.Y. (2017). The water footprint of wood for lumber, pulp, paper, fuel and firewood. Adv. Water Resour..

[28] (2010). Franklin Associates: Cradle-to-Gate Life Cycle Inventory of Nine Plastic Resins and Four Polyurethane Precursors. Report. 1. https://www.researchgate.net/publication/297268002_Cradle-to-Gate_Life_Cycle_Inventory_of_Nine_Plastic_Resins_and_Four_Polyurethane_Precursors.

[29] Boustead I. (2005). Eco-Profiles of the European Plastics Industry: Polypropylene (PP). PlasticsEurope. https://www.researchgate.net/publication/318864901_Eco-profiles_and_Environmental_Product_Declarations_of_the_European_Plastics_Manufacturers_-_Polypropylene_PP.

[30] Korol J., Hejna A., Burchart-Korol D., Wachowicz J. (2020). Comparative analysis of carbon, ecological, and water footprints of polypropylene-based composites filled with cotton, jute and kenaf fibers. Materials.

[31] Ferraro A., Camurati I., Dall’Occo T., Piemontesi F., Cecchin G. (2006). Advances in Ziegler-Natta catalysts for polypropylene. Kinet. Catal..

[32] Hossain M.T., Shahid M.A., Mahmud N., Habib A., Rana M.M., Khan S.A., Hossain M.D. (2024). Research and application of polypropylene: A review. Discov. Nano.

[33] Maddah H.A. (2016). Polypropylene as a Promising Plastic: A Review New organic semiconductor thin film derived from p-toluidine monomer View project Polypropylene as a Promising Plastic: A Review. Am. J. Polym. Sci..

[34] Kissel W.J., Han J.H., Meyer J. (2003). Polypropylene: Structure, Properties, Manufacturing Processes, and Applications. Handbook of Polypropylene and Polypropylene Composites.

[35] Bal B.C., Altuntaş E., Narlıoğlu N. (2023). Some selected properties of composite material produced from plastic furniture waste and wood flour. Mobilya Ve Ahşap Malzeme Araştırmaları Dergisi.

[36] Shahruzzaman M., Biswas S., Islam M.M., Islam M.S., Rahman M.M. (2019). Furniture: Eco-Friendly Polymer Composites Applications. Encyclopedia of Polymer Applications.

[37] Ichim M., Filip I., Stelea L., Lisa G., Muresan E.I. (2023). Recycling of Nonwoven Waste Resulting from the Manufacturing Process of Hemp Fiber-Reinforced Recycled Polypropylene Composites for Upholstered Furniture Products. Sustainability.

[38] Isaeva V.I., Aizenshtein É.M., Soboleva O.N. (1997). World production and use of polypropylene fibres and thread. A review. Fibre Chem..

[39] Kausar A. (2018). A review of filled and pristine polycarbonate blends and their applications. J. Plast. Film Sheeting.

[40] Bendler J.T. (1999). Handbook of Polycarbonate Science and Technology.

[41] Hafad S.A., Hamood A.F., Alsalihi H.A., Ibrahim S.I., Abdullah A.A., Radhi A.A., Al-Ghezi M.K., Alogaidi B.R. (2021). Mechanical properties study of polycarbonate and other thermoplastic polymers. J. Phys. Conf. Ser..

[42] Song P., Trivedi A.R., Siviour C.R. (2023). Mechanical response of four polycarbonates at a wide range of strain rates and temperatures. Polym. Test..

[43] Chang Y.W., Cheng J.H. (2012). Material characterization of polycarbonate near glass transition temperature. J. Chin. Inst. Eng..

[44] Rosato D.V. (2011). Consumer Products End Use Applications. Plastics End Use Applications.

[45] Antonenko J.S., Zhdanova N.S., Mishukovskaya J.I. (2021). Research Results of Possibility of Using Non-Traditional Materials in Design of Furniture for Children. IOP Conf. Ser. Mater. Sci. Eng..

[46] (2012). Furniture—Chairs and Tables for Educational Institutions Part 2: Safety Requirements and Test Methods.

[47] Ruiz-Mercado G.J., Smith R.L., Gonzalez M.A. (2012). Sustainability indicators for chemical processes: I. Taxonomy. Ind. Eng. Chem. Res..

[48] Ruiz-Mercado G.J., Smith R.L., Gonzalez M.A. (2012). Sustainability Indicators for chemical processes: II. Data needs. Ind. Eng. Chem. Res..

[49] Schyns Z.O., Shaver M.P. (2021). Mechanical recycling of packaging plastics: A review. Macromol. Rapid Commun..

[50] Coates G.W., Getzler Y.D. (2020). Chemical recycling to monomer for an ideal, circular polymer economy. Nat. Rev. Mater..

